# Wettability and adhesion of nanotubes applied to the surface of titanium implants by anodic oxidation

**DOI:** 10.1590/1807-3107bor-2024.vol38.0091

**Published:** 2024-09-09

**Authors:** Rogério de Lima ROMEIRO, Jorge Luiz ROSA, Lyncoln da Silva SIQUEIRA, Marcos GIOVANETTI, Davi Romeiro AQUINO, Patricia Fretes WOOD, Sandra Giacomin SCHNEIDER, Gustavo Grolli KLEIN

**Affiliations:** (a) Fundação Universitária Vida Cristã – Funvic, Discipline of Oral Surgery, Pindamonhangaba, SP, Brazil.; (b) Faculdade de Tecnologia de Pindamonhangaba – Fatec, Pindamonhangaba, SP, Brazil.; (c) Universidade de Taubaté – Unitau, Graduate Program, Taubaté, SP, Brazil.; (d) Centro de Ensino e Pesquisa Odontológica e de Nanotecnologia – Ceosp-Nanotec, Department of Nanotechnology, São Miguel do Oeste, SC, Brazil.; (e) Universidade del Pacífico, Discipline of Implant Dentistry, Asunción, Paraguay.; (f) Universidade de São Paulo – USP, School of Engineering of Lorena, Department of Materials Engineering, Lorena, SP, Brazil.; (g) Centro de Ensino e Pesquisa Odontológica e de Nanotecnologia – Ceosp-Nanotec, Department of Implant Dentistry, Chapecó, SC, Brazil.

**Keywords:** Dental Implants, Titanium, Surface Properties, Wettability

## Abstract

The aim of this study was to evaluate the wettability and adhesion of self-organized TiO_2_ nanotubes formed on the surface of 8 commercially pure titanium (CP-Ti) disks and 12 dental implants (n = 12) by anodization in a glycerol-H2O (50-50 v/v) electrolyte containing NH_4_F. Two disk specimens were not submitted to anodization (controls). The nanotubes thus obtained had average dimensions of 50 nm in diameter by 900 nm in length. The treated disk specimens were stored for 2, 14 and 35 days (n = 2), and the wettability of their surfaces was evaluated with a goniometer at the end of each storing period. The adhesion of nanotubes to titanium was evaluated by field emission scanning electron microscopy after subjecting the 12 implants to a simulation of clinical stress in two-part synthetic bone blocks. After installing the implants with the application of an insertion torque, the two halves of the block were separated, and the implants were removed. The nanotubes remained adhered to the substrate, with no apparent deformation. The contact angles after 14 days and 35 days were 16.47° and 17.97°, respectively, values significantly higher than that observed at 2 days, which was 9.24° (p < 0.05). It was concluded that the method of anodic oxidation tested promoted the formation of a surface suitable for clinical use, containing nanotubes with levels of wettability and adhesion to titanium compatible with those obtained by other methods found in the literature. The wettability, however, did not prove stable over the tested storage periods.

## Introduction

There is a consensus among researchers that the deposition of bone tissue on the surfaces of titanium implants takes place regardless of whether they are smooth or textured; however, roughness plays an important role in the percentage of bone apposition, promoting differences in the quality and speed of osseointegration.^1,2^

An appropriate surgical technique can provide favorable osseointegration, even in machined implants with no surface treatment;^2^ however, the use of implant surfaces with controlled roughness produces good results in graft sites, low bone density sites, or in patients with some systemic involvement. It also improves the predictability of results under normal bone conditions, by reducing the time required for osseointegration.

Rough surfaces allow implants to receive functional loads more precociously.^1^ For this to occur, it is important that surface treatment technologies be developed to improve the ability of implant surfaces to bond to bone. In addition, selection of electrochemically stable materials with adequate long-term corrosion resistance is essential.

Titanium is a metallic material whose biocompatibility is widely recognized owing to its ability to prompt the formation of an oxide layer on its surface. This protective layer, which forms passively upon contact with oxygen, prevents metallic ions from being released, thus preventing metal corrosion. Furthermore, when damaged, the oxide layer is immediately reconstituted within a very short period of time (2 ms) upon contact with an oxidizing medium, such as body fluid. Therefore, the living tissue around the implant is always in contact with this superficial oxide layer, and not with the substrate.^3^

Titanium oxide (TiO_2_) is the oxide that mostly forms from the reaction of titanium with oxygen at room temperature, and is also the most stable. Initially amorphous, it is a thin film that can become organized in a certain crystalline structure, as long as specific treatment and growth conditions are provided.

Techniques for modifying the titanium surface are also used to improve the adhesion of cells involved in the osseointegration of this material after implantation. In the last decades, methods of surface modification of dental implants have been developed to produce self-organized TiO_2_ nanotubes with the aim of providing improved osseointegration.^4^

According to Minagar et al.,^5^ when TiO_2_ films are in an environment such as body fluid, they generally induce the formation of superficial apatite layers. This is why osseointegration is accelerated, based on the chemical bond that forms between the material and the bone tissue. The methods used to induce the formation of TiO_2_ on titanium substrates include thermal oxidation, electrochemical anodization, the sol-gel process, thermal spray and chemical vapor deposition, among others.^6^

Anodization consists of applying a potential difference between the material to be anodized (anode) and a cathode, often made of platinum, both immersed in a specific electrolyte. It is a relatively simple method that allows better control of the diameter, thickness and length of the nanotubes.^7^

The growth of the anodic film is basically determined by obtaining a balance between the rates of formation and dissolution of the oxide film, considering the nature of the electrolyte, mainly determined by the presence of the fluorine ion. The anodic film growth process is also closely linked to the processing parameters, which include electrolyte concentration, applied potential, density of the current, time and pH.

**Figure 1:**
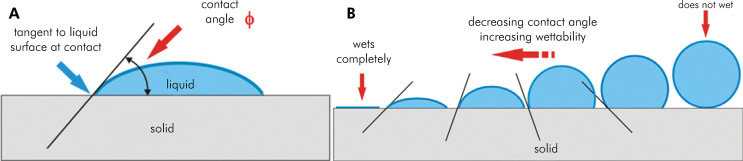
Schematic representation of the concept of wettability of a surface. A: Contact angle measurement. B: Correlation between contact angle and wettability.

The wettability of the surface—quantified by the contact angle between a liquid and a solid substrate—is one of the parameters that most greatly affect the biological response to an implanted biomaterial, affecting protein adsorption, platelet adhesion/activation, blood coagulation, and cell adhesion.^8^ Figure 1 shows a schematic representation of the contact angle measurement as a parameter of the wettability property of a surface.

A little more than a decade ago, many research studies were devoted to the study and characterization of self-organized, highly ordered nanotubes formed by electrochemical anodization, as a surface modification technique for Ti and its alloys, used in biomaterial applications. There is a wide range of chemical media used for this purpose, such as solutions of H_2_PO_4_, HF, H_2_SO_4_, Glycerol, NaF and others,^9^ used in electrochemical processes with different sets of parameters.

Aiming at promoting improved osseointegration, Rosa et al.^10^ studied the main parameters involved in the formation of nanotubes during the electrochemical anodization process of a grade IV, commercially pure titanium (CP–Ti) alloy, and established a set of conditions that promoted a high percentage area of TiO_2_ nanotubes, and optimal surface morphology quality.

Previous studies have researched different methods used to obtain nanotubes on the surface of implants;^11^ however, to the best of our knowledge, no previous work has investigated the adhesion of the nanotubes formed by these methods in two-part synthetic bone blocks, so as to eliminate the confounding factor represented by the pre-assessment application of a counter-torque to remove the implants from their substrate.

The aim of the present study was to evaluate the wettability (as measured by the contact angle), the influence of storage time on wettability, and the adhesion of nanotubes formed on the flat surface of grade IV CP-Ti test specimens, and on the machined surfaces of commercial screw-shaped dental implants, by the anodic oxidation method, after application of an insertion torque in two-part blocks of synthetic bone. The method of electrochemical anodization used for the growth of nanotubes on the Ti surface was the same as that described by Rosa et al.^10^

## Methods

In order to establish the number of study specimens, a sample size calculation was performed based on data previously collected from the literature. A significance level of 95% and power of 80% were adopted. Based on this calculation, 12 external hexagon implants measuring 3.75 x 13 mm and 8 flat disks measuring 12 x 3 mm were used. Both the implants and the disks were made of grade IV CP-Ti, with machined surfaces. All the materials were supplied by SIN - Sistema de Implante Nacional (São Paulo, Brazil).

The disks were used to evaluate the wettability of the anodized surfaces by measuring contact angles. Their simpler structure, compared to that of implants, allowed more accurate measurement of wettability, as well as a comparison of our study results with those of other studies that used the same specimen type. The implants were used to asses the adhesion of the nanotubes formed on the anodized surface. Both specimen types were evaluated under SEM.

Two disks were used for each experimental time period, namely 2, 14 and 35 days. Two disks were not submitted to the experimental treatment, and served as controls (machined only). After each experimental time period, one treated disk was submitted to SEM for visual evaluation of the nanotubes, and one was submitted to the wettability test.

For the cleaning step, each specimen was fastened to a titanium rod and then placed in an analytical ultrasound basin (Usina Ind. Com. e Importação, Santo Ângelo, Brazil) in an alcohol medium (96% GL) for 30 min. The specimen was then dried in an argon stream and placed in the electrolytic solution.

The same conditions used by Rosa et al.^10^ for the electrochemical anodization procedure were used in this study, namely 50% (v/v) hydrated glycerol (glycerol-H_2_O) in a deionized water solution and 1% (by mass) ammonium fluoride (NH_4_F), to which a voltage of 20 V and current of 3 A was applied for 2 h.

For the anodization step itself, a simple experimental setup was made with two electrodes in a 500 mL electrolytic cell containing a volume of 150 mL of the electrolytic solution, where the anode was the test specimen and the cathode was a platinum counter-electrode. A magnetic stirring plate (Fisatom Equipamentos Científicos, São Paulo, Brazil) was used to homogenize the electrolyte.

A voltage and current stabilizer (Matsusada Precision, San Jose, USA) was set to apply 20 V and 3 A. After 2 h, the specimen was removed and dried in an argon stream. The electrolytic solution was renewed after the anodization of 3 specimens, and the platinum counter-electrode was cleansed with deionized water after each specimen change.

The flat-surface test specimens were used to read the contact angle between nanotubes and substrate (wettability). The screw-shaped implants were used for the adhesion test.

### Wettability assessment

In order to assess the influence of time on wettability, the disk specimens were submitted to different storage periods, namely 2, 14 and 35 days. Three drops of deionized water, each containing 0.5 to 0.75 μL, were placed on each specimen. Nine measurements were made for each experimental time period with a goniometer (model 290, Ramé-hart Instrument Co., Succasunna, USA). This instrument uses a computer program to measure the angle formed between a drop of water and a given surface. The contact angles were obtained for each drop, and then the contact angle mean and standard deviation values were calculated for each specimen.

### Adhesion assessment

**Figure 2:**
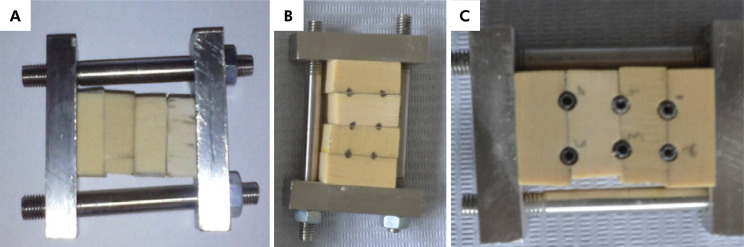
A: Device used to simulate implant placement. B: Marking the perforations to be made in the slots between blocks. C: Installed implants.

The screw-shaped machined implants were installed in two-part synthetic bone blocks (Block 10 PCF, SKU1522-01; Sawbones, Vashon Island, USA). Previous studies conducted to investigate the adhesion of nanotubes to their substrates^12,13,14^ used bovine bone or synthetic bone;^15^ however, because the test implants would have to be removed to evaluate their surface under the microscope, a bone block supporting device was developed, so that the two parts of the block could be separated after implant placement, and the implants could be removed without requiring the application of a counter-torque, thus simulating a condition closer to the clinical reality. Two devices were selected, and 6 holes were drilled, two in each slot between blocks, following the drilling sequence recommended by the manufacturer (Figure 2).

**Figure 3:**
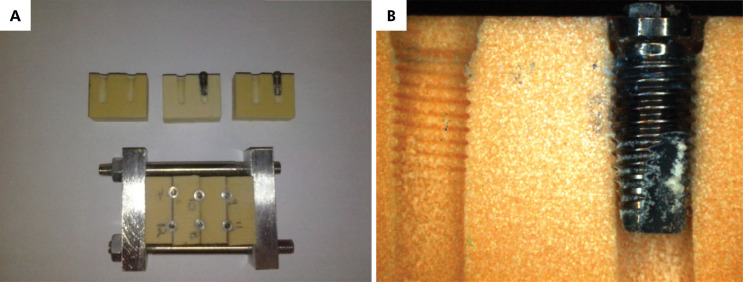
A, B: Device with artificial bone and implants.

A total of 12 implants were placed randomly (www.randomizer.org), 6 in each supporting device. The insertion torque applied was measured with a digital torque wrench (Mark-10 Corporation, Copiague, USA). After insertion of the implants, the device was disassembled, and the implants were removed and sent to be analyzed under a field emission scanning electron microscope (SEM-FEG; Phillips XL-30; Crawley, West Sussex, UK) employing a working distance of 2–6 mm and voltage of 5–20 KV, in SE mode. Figure 3 shows the appearance of the blocks after opening the device and removing the implants.

**Figure 4:**
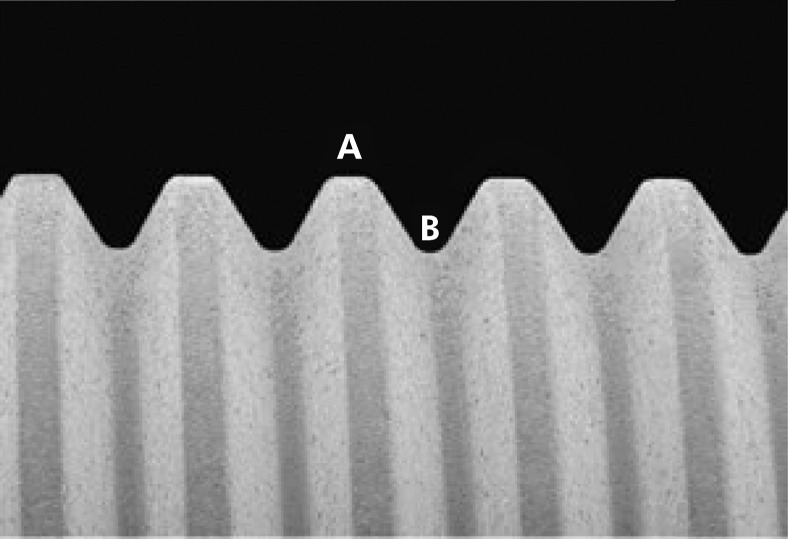
Illustration of the regions of a machined-surface implant after the application of nanotubes, analyzed using FESEM. Region A, crest of the thread, and Region B, valley of the thread.

After specimen metallization, each implant was divided into two assessment regions: Region A, the crest of the thread, and Region B, its valley (Figure 4).

### Statistical analysis

The paired t-test was used to compare the contact angles observed after each experimental time period. BioEstat 5.0 and SPSS 13.0 software was used in the statistical analysis of the data. A descriptive analysis was performed of the torque values applied for implant placement in preparation for the nanotube adhesion assessment.

## Results

### Surface characterization

**Figure 5:**
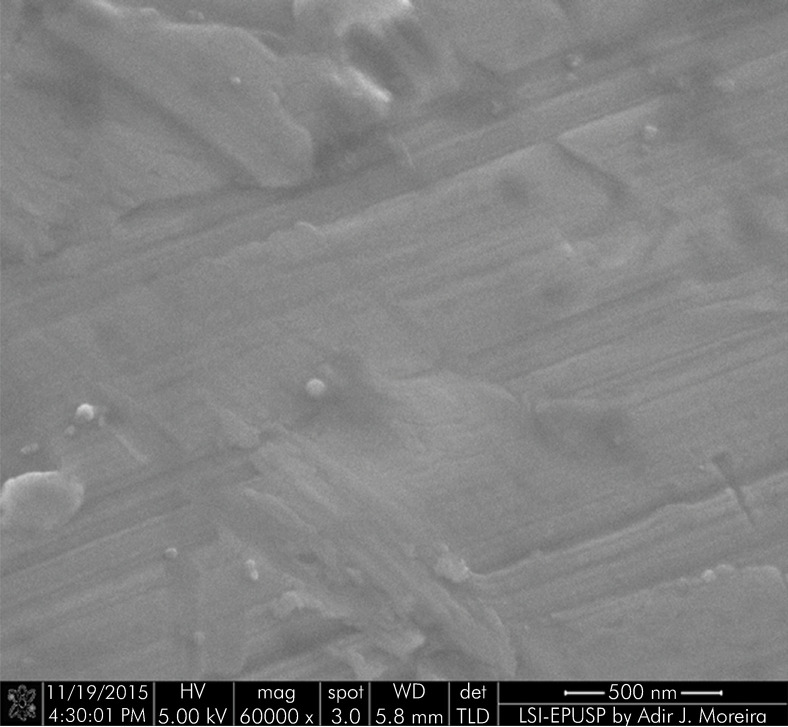
FESEM image of the surface of a machined implant without the application of nanotubes (60,000x).

**Figure 6:**
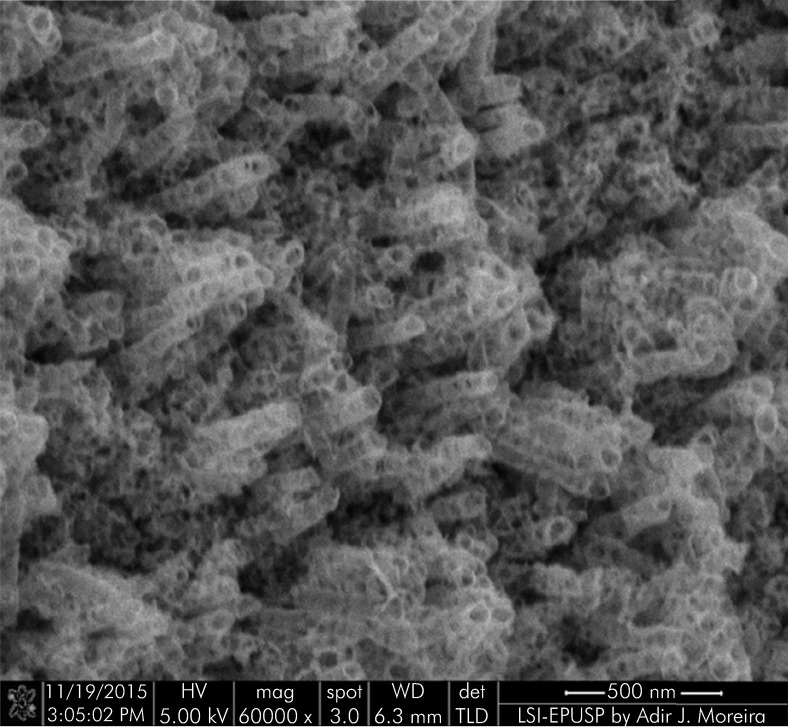
FESEM image of the surface of an implant after the application of nanotubes (60,000x).

Figure 5 and 6 show the topography of a machined-surface and a surface-modified implant, respectively, at 60,000x magnification. The modified surface of the implant is morphologically homogenous and covered by perpendicularly arranged nanotubes.

### Wettability

The contact angles observed in both treated and untreated disk specimens between the nanotubes and the titanium substrate are shown in Table 1. Two days after specimen preparation, the contact angle measurements of the CP-Ti modified-surface were significantly lower than those observed on the surface of the untreated CP-Ti (p < 0.05). However, the contact angles observed after 14 and 35 days were significantly higher than that observed after 2 days (p < 0.05).

**Table 1:** Mean and standard deviation values of the contact angle measurements of CP-T test specimens subjected to anodic oxidation, according to storage time.

	Without anodization	Storage time		
		35 days	14 days	2 days
Contact angle (°)	88.14^(A)^ ± 3.81	17.97^(B)^ ± 2.42	16.47^(B)^ ± 1.32	9.24^(C)^ ± 0.47

Different letters indicate a statistically significant difference according to Student’s paired-samples t-test (p < 0.05).

### Adhesion

**Figure 7:**
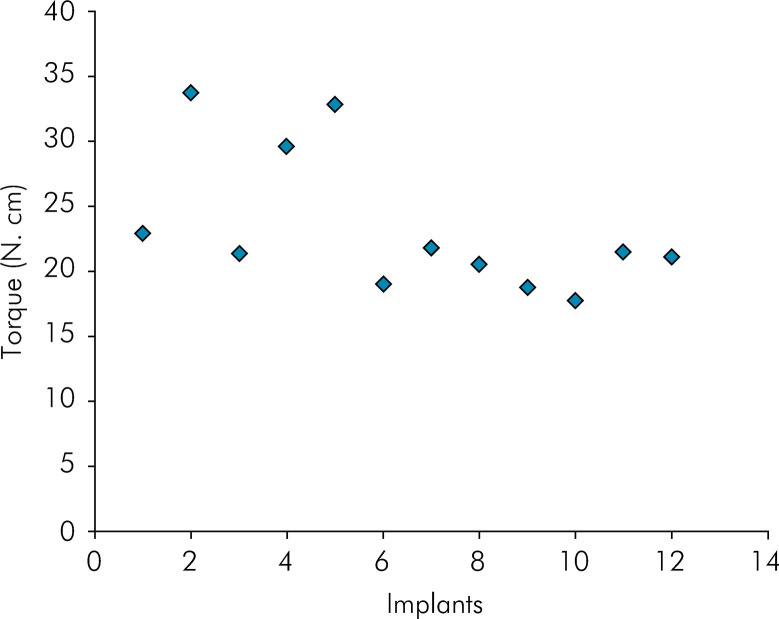
Insertion torque values of grade IV CP-Ti implants with self-organized nanotubes formed on their surface.

**Figure 8:**
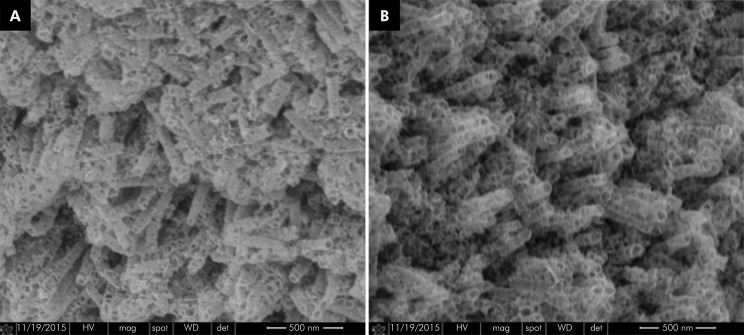
FESEM image of the machined surface of a grade IV titanium implant subjected to anodization with nanotubes. A: Regions A, crest of the thread. B: Region B, valley of the thread (60,000x).

The insertion torque values of the 12 implants installed are shown in Figure 7. The mean torque applied was 23.3 N.cm, a value compatible with clinical reality. In both the implant regions (A, crest, and B, valley), there were no distorted or detached nanotubes (Figure 8).

## Discussion

The present study examined the wettability and adhesion of an anodic film of nanotubes applied to grade IV CP-Ti machined implants. The results demonstrated that the formation of nanotubes on the test disks was similar to that observed in the research conducted by Rosa et al.,^10^ who performed the same anodic oxidation process using smooth-surface implants.

The nanotubes formed on the crests of the implant threads were more irregular, probably owing to the marked change in surface angulation, whereas the nanotubes formed in the less angled surfaces were more regular.

After forming nanotubes on the grade IV CP-Ti disks, surface wettability—the main surface feature responsible for accelerating osseointegration^16,17^—was assessed. According to Duncan et al.,^18^ a reduced contact angle is decisive for speeding up osseointegration. It is generally believed that an increase in surface roughness results in an increase in the contact angle. However, in a study by Lemons,^16^ specimens treated to provide greater roughness exhibited significantly lower contact angles than polished specimens, although there was no significant difference between their roughness measurements, indicating that a rough surface is not enough; it needs to have high wettability.

The wettability of the surface is one of the parameters that most greatly affect the biological response to an implanted biomaterial, influencing protein adsorption, platelet adhesion/activation, blood coagulation, and cell adhesion.^8^ The study of the molecular interaction between two fluids (e. g., liquid and vapor) and a surface is made by measuring the so-called contact angle (ϴ). This is defined as the angle between a plane tangent to a drop of the liquid and the plane containing the surface where the drop is deposited.

In the present study, the selection of implants without prior treatment to undergo anodic oxidation was based on studies such as that of Kilpadi et al.^19^ Comparing the wettability results of the smooth specimens with those of the specimens submitted to the oxidation treatment, the authors concluded that a smooth surface is more hydrophobic, and should therefore induce a slower osseointegration process.

The results showed that, initially, the anodization was extremely positive with respect to CP-Ti wettability. The loss of this condition over time suggests absorption of the humidity of the environment itself by capillary movement of fluids into the nanotubes, causing the surface to have a hydrophobic behavior. This observation reinforces the need for adequate storage of implants prior to their use.

The ability of the nanotubes to adhere to the surface of grade IV CP-Ti was evaluated in the present study after submitting them to the mechanical stress resulting merely from inserting the implant into the bone. Since the last bone drill has a diameter 0.5 mm smaller than that of the implant, an insertion torque between 10 and 35 N.cm is applied to provide the implant with minimum primary stability, a factor considered essential for osseointegration.^20^

Previous studies conducted to investigate the adhesion of nanotubes to their substrates^12,13,14^ used bovine bone or synthetic bone^15^; however, comparison of their results with those of the present study is impaired because, in those studies, the implant was installed with an insertion torque and then removed with a counter-torque, a procedure that renders the experimental model far removed from clinical reality. For this reason, a device was developed in the present study, in which the bone block simulating implant installation could be separated into two halves, so as to allow the implant to be removed without the application of any counter-torque. As a result, images were obtained showing the nanotubes that were retained on the entire surface of the implant, thus demonstrating—without the interference of a factor extraneous to clinical reality—that the anodic oxidation process used promoted adequate levels of adhesion of the nanotubes to the titanium substrate of the implants tested.

## Conclusions

The proposed electrochemical anodization technique proved effective in producing the formation of TiO_2_ nanotubes, both on disks and on screw-shaped implants made of grade IV CP-Ti treated with double acid etching.

The TiO_2_ nanotubes obtained presented a degree of hydrophilicity adequate for their clinical use.

The TiO_2_ nanotubes obtained were adhered to the titanium substrate, even after subjecting the implants tested to an installation torque in synthetic bone blocks.
